# CD4:CD8 ratio as a frontier marker for clinical outcome, immune dysfunction and viral reservoir size in virologically suppressed HIV-positive patients

**DOI:** 10.7448/IAS.18.1.20052

**Published:** 2015-06-29

**Authors:** Wei Lu, Vikram Mehraj, Kishanda Vyboh, Wei Cao, Taisheng Li, Jean-Pierre Routy

**Affiliations:** 1 Chronic Viral Illnesses Service, McGill University Health Centre, Montreal, Quebec, Canada; 2 Research Institute of the McGill University Health Centre, Montreal, Quebec, Canada; 3 Division of Infectious Diseases, Peking Union Medical College Hospital, Beijing, China; 4 Division of Hematology, McGill University Health Centre, Montreal, Quebec, Canada

**Keywords:** CD4:CD8 ratio, HIV infection, immune dysfunction, inflammation, viral reservoir

## Abstract

**Introduction:**

Absolute CD4 T cell count and plasma viral load have been established as predictors of HIV disease progression, and CD4 T cell count is used as an indicator for initiation of antiretroviral therapy. Following long-term therapy, patients generally present with significant CD4 T cell recovery contrasting with persistently elevated CD8 T cell counts, which leads to a partial restoration of CD4:CD8 ratio. This review focuses on the relevance of the CD4:CD8 ratio on clinical outcomes, immune dysfunction and HIV reservoir size in long-term treated patients.

**Method:**

We conducted a comprehensive literature review of publications in English language using major electronic databases. Our search was focused on factors contributing to CD4:CD8 T cell ratio and clinical outcome in adult HIV-positive patients in the context of treated infection.

**Discussion:**

Low CD4:CD8 ratio has been linked to ageing and acts as a predictor of mortality in the general population. This ratio may represent the combined effects of inflammation and immunological changes called “inflammaging.” Although the mechanisms underlying partial correction of the CD4:CD8 ratio and persistently elevated CD8 T cell count in long-term treated patients remain poorly understood, it has been recently indicated that patients with optimal CD4 T cell recovery and low CD4:CD8 ratio still harbour increased immune activation, an immune senescent phenotype and have a higher risk of non-AIDS morbidity and mortality. This review reconsiders CD4:CD8 ratio in the light of advances in the understanding of immune dysfunction and examines its pathophysiological features and implications on clinical outcome and HIV reservoir size in long-term treated HIV-positive adults.

**Conclusion:**

The CD4:CD8 ratio can contribute to the immunological evaluation of treated patients in a long-term follow-up and may be applied for monitoring both immune dysfunction and viral reservoir size in immune-based clinical trials.

## Introduction

HIV infection is characterized by profound CD4 T cell destruction, compromised mucosal barrier function and chronic immune activation. In addition, this infection is associated with a marked activation and expansion of HIV-specific and bystander CD8 T cells [1]. The impairment in CD4 T cell regeneration and the persistent elevation of CD8 T cell counts are considered to be a consequence of viral persistence and multiple inflammatory factors including gut microbial translocation, leading to major T cell dysfunction [2,3]. Antiretroviral therapy (ART) in a majority of patients suppresses HIV plasma viral load (VL) and stops the progression to AIDS, allowing progressive CD4 T cell recovery paired with a persistent elevation of CD8 T cells. Such changes on T cell populations over time result in a partial restoration of the CD4:CD8 ratio. Patients on suppressive ART who present with lower CD4:CD8 ratios have a higher risk for non-AIDS morbidity and mortality even with optimal CD4 T cell recovery [4]. However, the reason for the persistence of elevated CD8 T cell counts during HIV infection was reviewed but has not been elucidated [5].

This review focuses on the relationship between CD4:CD8 ratio and clinical outcomes, inflammation, as well as ageing and HIV reservoir size in the context of treated infection. We believe that the data reviewed here supports that the CD4:CD8 ratio represents a marker of immune dysfunction and may contribute to better patient management. Furthermore, this ratio may be helpful in allocating patients and assessing immune changes in immune-based clinical trials.

## Methods

A comprehensive review of the English-language publications was implemented. We searched PubMed, JSTOR and Scopus electronic databases with keyword combinations including CD4:CD8 ratio, CD4 T cell, CD4 T cell reconstitution/restoration/recovery, CD8 T cell persistence/elevation and ageing. The same strategy was used with Google Scholar and ISI Proceedings to further include non-peer-reviewed literature and conference publications. We also considered the number of citations and study sample size of the relevant papers for their selection and description in the text and/or tables. Our search was limited to adult HIV-1-positive subjects using publications from year 2000 to 2015 at a time when advances in ART have allowed long-term control of viral replication.

## Results and discussion

### Historical perspective of the CD4:CD8 ratio as a prognostic factor for disease progression

In 1989, Taylor *et al*. compared the role of three immunological parameters in predicting disease progression to AIDS among 813 untreated HIV-seropositive men during a three-year longitudinal study, i.e. absolute CD4 T cell count, CD4 T cell percentage and the CD4:CD8 ratio. They found that these three parameters were strongly correlated to each other and showed comparable ability to predict the development of AIDS [6]. Older patient age can be used as a predictor of clinical events, so do the additional markers such as neopterin (a product of stimulated macrophages), β2-microglobulin, soluble interleukin-2 receptors (sIL-2R) and immunoglobulin A (IgA). However, among all these aforementioned markers, CD4 T cell count and the CD4:CD8 ratio stood out as the two best predictors for the progression to AIDS [7]. Level of CD8 T cell activation as measured by CD38 and HLA-DR expression is also an independent predictor of HIV disease progression [8,9]. Interestingly, elevated CD8 T cell counts and their level of activation represent independent factors associated with disease outcome.

Although the CD4:CD8 ratio has been used as a parameter to predict outcome [10–12], absolute CD4 T cell count is still considered the key criterion for ART initiation as it represents the best surrogate marker for the risks of opportunistic infections, AIDS-related cancers and death.

### The CD4:CD8 ratio acts as a surrogate marker of T cell compartment balance: CD4 T cell recovery and CD8 T cell expansion

**Figure 1:**
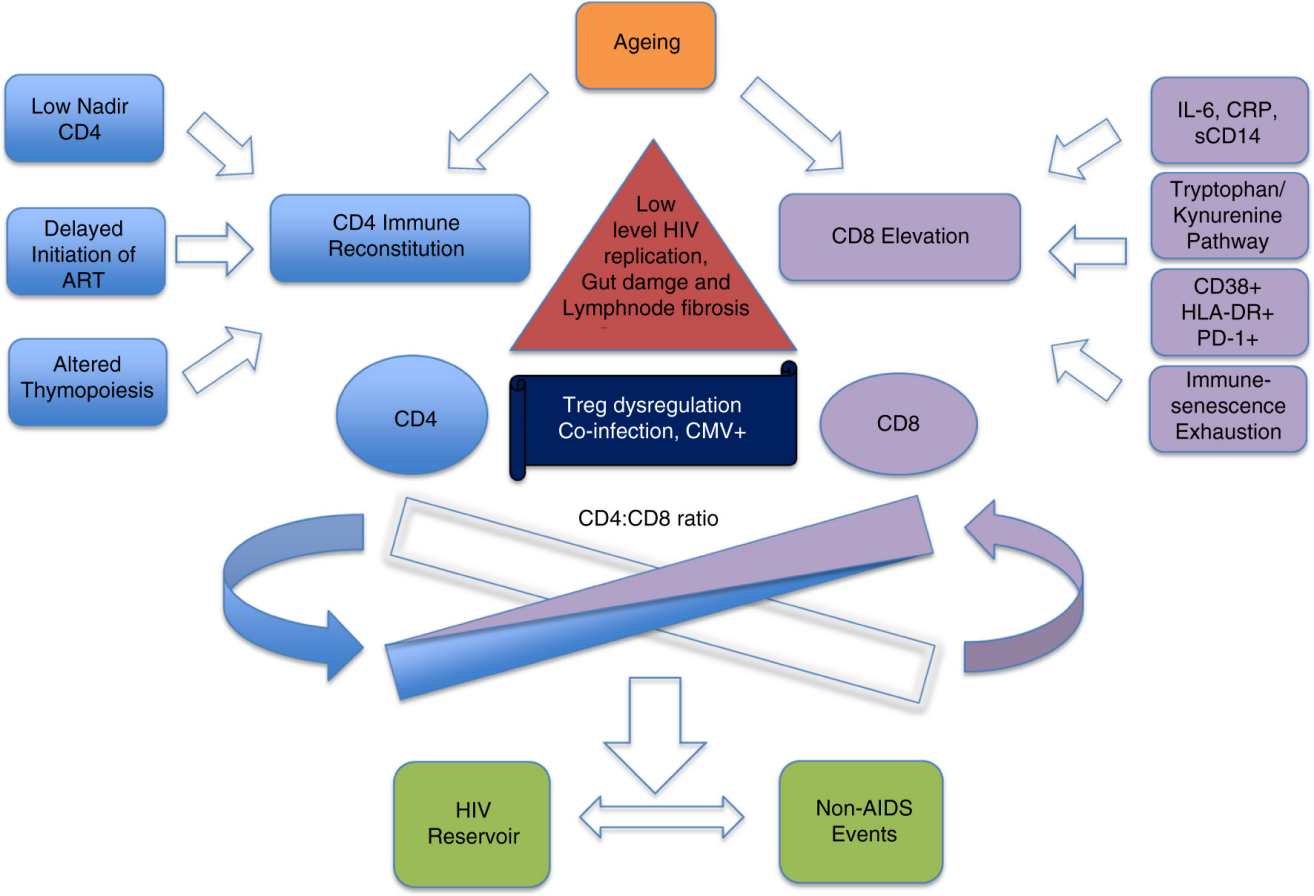
Factors associated with low CD4:CD8 ratio in HIV treated infection.

Long-term ART has successfully restored CD4 T cell counts in a large proportion of HIV-positive patients. However, the majority of these patients still demonstrate a persistent elevation of CD8 T cell count as well as dysfunction of CD8 T cell compartments [13]. The balance between immune reconstitution and immune activation/inflammation may be involved in the trend towards the normalization of the CD4:CD8 ratio with ART (Figure 1).

### The CD4:CD8 ratio foretells CD4 T cell recovery with long-term ART

CD4 T cell recovery is a sign of immune improvement following control of viral replication with ART [14]. The attainment of a CD4 T cell count over 500 cells/mm^3^ has been associated with a reduction in mortality [15]. Factors including gender, low pretreatment absolute CD4 T cell count (nadir T cell count), presence of CXCR4 (X4) tropic virus, altered thymopoiesis, overexpression of genes involved in immune activation and apoptosis and inflammation have been clearly identified as factors influencing CD4 T cell recovery [16–20]. Specifically, low CD4:CD8 ratio prior to initiation of ART was associated with a decreased probability of achieving CD4 T cell counts >500 cells/mm^3^ [4,21].

Serrano-Villar *et al*. have reported on the factors associated with a low CD4:CD8 ratio in HIV-positive adults from four distinct clinical cohorts and three clinical trials. They showed that a low CD4:CD8 ratio despite effective ART and CD4 T cell recovery above 500 cells/mm^3^ was associated with a skewed T cell phenotype from naïve towards terminally differentiated CD8+ T cells, with higher levels of CD8+ T cell activation (HLADR+CD38+), senescence (CD28- and CD57+CD28-) and higher kynurenine/tryptophan ratio [13]. Most significantly, by using multiparametric bioinformatics, Buggert *et al*. identified a correlation between the CD4:CD8 ratio and CD4 T cell populations that over-expressed activation and exhaustion markers such as programmed death-1 (PD-1) [22]. Equally, the CD4:CD8 ratio displayed a significant correlation with absolute CD4 T cell recovery during a two-year treatment period. Interestingly, compared with the absolute CD4 T cell count and HIV plasma VL, the CD4:CD8 ratio before ART initiation acted as a much better predictor for CD4 T cell recovery.

Ndumbi *et al*. monitored the longitudinal changes in CD4:CD8 ratio over a 10-year follow-up in ART treated patients [23]. They demonstrated that in patients with a CD4 T cell count<200 cells/mm^3^, the CD4:CD8 ratio failed to normalize. Importantly, they found that CD4:CD8 ratio normalization resulted from the combination of an optimal increase in CD4 T cell count with a concomitant decrease in CD8 T cell count. However, this only occurred in patients having a baseline CD4 T cell count>350 cells/mm^3^. Conversely, in patients with CD4 T cell counts<350 cells/mm^3^, CD8 T cells remained elevated, a possible result of persistent residual low-level HIV replication, gut damage associated with immune activation, lymph node fibrosis, coinfections, immunosenescence and Treg dysregulation [24]. The determinants of CD4 recovery in the context of treated infection have been recently reviewed [25] (Figure 1).

A recent prospective observational study by Mussini *et al*. [26] on 3236 participants with a median CD4:CD8 ratio of 0.39 before ART reported the estimated normalization probability of 4.4, 11.5 and 29.4% after 1, 2 and 5 years of treatment, respectively. Higher CD4 T cell counts and higher CD4:CD8 ratio at baseline as well as absence of cytomegalovirus (CMV) serology have been more likely associated with ratio normalization.

### Persistence of expansion of CD8 T cells with skewed differentiation phenotype

**Figure 2:**
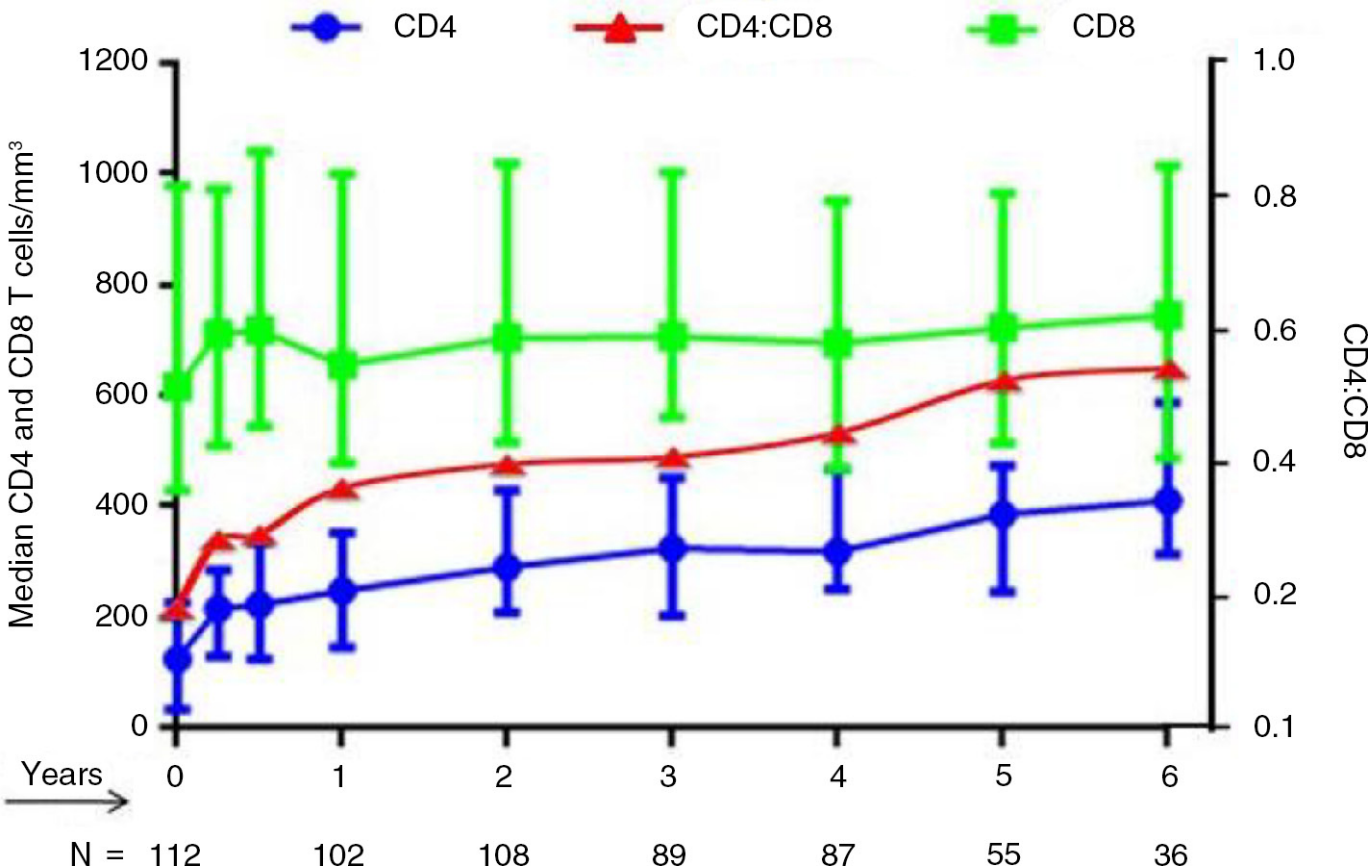
Trajectories of CD4 and CD8 T cells and CD4:CD8 ratio in long-term treated patients. (Advanced patients initiating ART, enrolled in Beijing cohort.)

Following effective long-term ART, about 50 to 80% HIV-positive patients achieve CD4 T cell counts >500 cells/mm^3^ depending on their baseline values and time on ART [27]. However, there are continuous quantitative, qualitative and functional defects in CD8 T cell compartment, resulting in low CD4:CD8 ratios. Recently Helleberg *et al*. examined the trajectories of CD8 T cells before and after ART in 3882 HIV-positive patients [24]. Their results showed that CD8 T cells were elevated during untreated HIV infection and remained elevated through 10 years of ART. This finding is concordant with our cohort of 109 HIV-positive late presenters, who harboured a CD4:CD8 ratio of 0.66 (0.19–1.84) after a median follow-up of 4.5 years on ART, with a median baseline CD4:CD8 ratio of 0.15 (0.01–0.76) (Figure 2, unpublished data).

Emu *et al*. also found that the increased CD4:CD8 ratio in ART-suppressed subjects compared to non-controllers was driven by an increase in CD4 T cells, contrasting with continuously expanded CD8 T cells [28]. Expansion of differentiated CD8 T cell subpopulations (CD28-CD27-CD45RA+/CCR7-) persisted despite ART and minimal changes were noted in naïve T cell counts over time. Increased numbers of exhausted CD8+CD28- T cells were associated with a low CD4:CD8 ratio. Those persistently expanded CD8 T cells in treated HIV-positive patients exhibit characteristics of T cell immune-senescence and exhaustion. Whether those expanded CD8 T cells persist durably without continued cell division remains to be determined [29]. These factors might contribute to the failure of the majority of patients receiving ART to achieve normalized CD4:CD8 ratios.

### Correlation of the CD4:CD8 ratio with immune activation and inflammation

The CD4:CD8 ratio is considered a marker for both immune-senescence and immune activation, even in patients with long-term suppressive viral control [30,31]. (Studies are depicted in Table 1.) Historically inflammation was regarded as a passive pathological consequence of injury and infection. Nowadays, it has been gradually considered also as a mechanism of immune defense and repair. Immune activation usually encompasses changes in cellular markers and soluble factors associated with inflammation [32], while inflammation refers more often to increases in soluble biomarkers such as interferon alpha (IFN-α), tumour necrosis factor alpha (TNF-α), IL-6 [33,34] and other cytokines and chemokines. Furthermore, the exhaustion marker PD-1 which regulates T cell activation and sCD14, a marker of monocyte/macrophage activation, has also been implicated in HIV pathogenesis [35–38].

**Table 1:** Studies on association of CD4:CD8 ratio with immunological and clinical outcome in chronically treated HIV patients

							Correlations			
							
Reference	Patients treated/untreated	Number of patients (N)	Duration of ART (years)	CD4: CD8 ratio criteria	Normalization of CD4:CD8 percentage	Factors associated with normalization of CD4:CD8 ratio	Activation HLA-DR+ CD38+	Immuno- senescence CD57+CD28-	Inflammation IL-6, hs-CRP, sCD14	Non-AIDS morbidity and mortality
[46]	Treated HIV- RNA<50	4206	2.7	>1.2	7.2%	Baseline CD4>350, CD8<500,CD4:CD8>0.5	NA	NA	NA	No
[13]	Treated with viral suppression and CD4≥500 cells/mm^3^	C1>1500 C2>2200 C3=122 C4=2400	Different durations	≤0.4 defined as low	NA	Positively correlated with T_N,_ T_CM_ and T_TM_ and negatively correlated with T_EM_.; predictor of the K/T	Yes	Yes	Yes	Yes
[30]	Treated 76% undetectable	112	>15	≥0.9	37%	Older age, nadir CD4 and detectable HIV VL associated with inverted CD4:CD8 ratio	Yes	Yes	No	No
[23]	Treated HIV-RNA<50	288	>10	>1.2	16% (most in baseline CD4>350)	Failure to normalize the complete T cell phenotype when delaying cART with a CD4<200	NA	NA	NA	NA
[4]	Treated	407	4	Low <0.4			NA	NA	NA	Yes

VL= viral load; C=Cohort; ART=antiretroviral therapy; K/T=Kynurenine/Tryptophan; NA=Not applicable. T_N_=Naïve T cells; T_CM_=Central; Memory T cells; T_TM_=Transitional Memory T cells; T_EM_=Effector Memory T cells.

A recent cross-sectional study reported association of CD4:CD8 ratio with factors that include CD4 nadir, pre-ART plasma VL, duration of ART, level of expression of HLADR+CD38+ on CD4 T cells and CD57+ on CD8 T cells. CD4:CD8 ratio remained independently associated with T-cell activation [31].

Immune activation has been linked with CD4:CD8 ratio as reported by Sainz *et al*. in vertically infected children and adolescents where they correlated CD4:CD8 ratio and CD4 T cell percentage with immune activated (CD38+HLA-DR+) and senescent (CD28-CD57+) CD4 and CD8 T cells [39]. An inverted CD4:CD8 ratio was associated with higher frequencies of activated, senescent CD4 and CD8 T cells, and a skewed T-cell phenotype from naive towards effector memory. Furthermore, the CD4:CD8 ratio was strongly correlated with CD8 T cells expressing exhausted phenotype (HLA-DR+PD-1+).

The similar correlation between low CD4:CD8 ratios, altered T cell subsets and elevated CD8 T cell activation was reported by Serrano-Villar *et al*. in ART-treated patients demonstrating CD4 T cell counts>500 cells/mm^3^ [13]. In addition, they observed an inverse correlation between the CD4:CD8 ratio and hs-CRP, IL-6, and markers of myeloid cell activation such as sCD14 and indoleamine 2,3-dioxygenase (IDO). IDO is an immune-modulatory enzyme, which breaks down tryptophan into kynurenine and can be considered as a myeloid cell marker of immune suppression [40,41]. For patients with CD4 T cell counts >500/mm^3^, only the tryptophan/kynurenine (KT ratio), a marker of IDO activity, remained significantly correlated with the CD4:CD8 ratio and was able to predict non-AIDS events. These important findings sparked a renewed interest in the association of the CD4:CD8 ratio with the KT ratio, which has been recognized as a key factor contributing to HIV immune dysfunction [42,43]. Taken together, the correlation of CD4:CD8 ratio with IDO and KT ratio indicates that myeloid cells might play a more important role in immune activation than lymphoid cells in long-term ART treated patients [44,45].

### The physiological CD4:CD8 ratio and ageing with its implications in HIV infection

In the general population a CD4:CD8 ratio of less than 1.0 is considered as a surrogate marker of immunosenescence and represents an independent predictor of overall mortality [47]. Convergent evidence indicates that a gradually declining CD4:CD8 ratio correlates with immune dysfunction leading to a poor response to immunization, as well as increased risks of severe infections and malignancies compared to younger individuals in the general population [48,49].

The characteristics of age-associated inflammation, termed “inflammaging,” are very similar to inflammatory changes that occur during HIV infection [50]. Our group recently reported that CD4:CD8 ratio was 2.11±0.99 in uninfected healthy adults and spanned from 0.70±0.47 in recently infected patients to 0.1±0.05 in very advanced patients [42,51]. This ratio gradually increased up to 0.94±0.37 after an average of nine years of ART. Interestingly, elite controllers, who are able to maintain an undetectable VL in absence of ART presented with a relatively low CD4:CD8 ratio when compared to the uninfected control subjects (1.13±0.59 vs. 2.1±0.99 respectively) [42]. We assessed ageing effect by a cross sectional analysis on 893 successfully treated HIV-positive patients with undetectable VL for at least one year, the CD4:CD8 ratio significantly lower in patients in their eighties when compared to those in their twenties 0.7 vs. 1.0, respectively (*p*=0.014*) (unpublished data). Of interest, we also note that male patients had lower CD4:CD8 ratio than females independent of age and duration of treatment. Inverted CD4:CD8 ratio together with loss of both naïve CD4 and CD8 T cells, expansion of activation and senescence markers on CD4 and CD8 T cells are presented both in HIV infection and ageing [52]. Differences in T cell compartments may echo changes observed in premature ageing associated with HIV infection [53]. The relationship of chronic inflammation, aberrant T cell function and phenotype as related to biologic ageing in HIV infection needs further investigation.

### Low CD4:CD8 ratios predict non-AIDS related morbidity and mortality in treated HIV infection

Several studies reported a predictive value of CD4:CD8 ratio on clinical outcomes in treated patients. However, the cutoff values of CD4:CD8 ratio used in these studies are not consistent, ranging from 0.8 to 1.5. The ratio used in these studies may depend according to the sample size, nadir CD4 T cell count, timing and duration of ART. Despite this inconsistency in defining a cutoff of CD4:CD8 ratio, these studies still generate important information on its predictive value for clinical outcomes, as depicted in Table 1.

The relationship of the CD4:CD8 ratio and non-AIDS related mobility and mortality was conflicting. Leung *et al*. studied 4206 patients for a median follow-up of 2.7 years and identified 306 individuals (7.2%) who demonstrated normalized CD4:CD8 ratios (≥1.2) [46]. By using the Kaplan-Meier curves of the time to CD4:CD8 normalization, the probability of achieving a normal CD4:CD8 ratio was 6.1% for those with baseline CD4 T cells<200 cells/mm^3^, compared to 21% for those with CD4 T cells >350 cells/mm^3^. Interestingly, no plateau was reached; suggesting that the increase of CD4:CD8 ratio may continue. They found that low CD4 T cell counts, old age and intravenous drug use (IDU) were risk factors that were significantly associated with increasing risk of non-AIDS-events.

The relationship between CD4:CD8 ratio and non-AIDS events, was investigated in a case-control study performed by Serrano-Villar *et al*. [4]. Multivariate analyses adjusted for age, sex, nadir CD4, proximal CD4 T cell count, year of ART initiation and ART duration were performed on 407 patients for the prediction of non-AIDS events, including malignancies, cardiovascular and kidney diseases. A low CD4:CD8 ratio was an independent factor for both non-AIDS morbidity and mortality in long-term treated HIV-positive patients and was independent of nadir CD4 T cell count. Encouragingly, Saracino *et al*. reported that patients with more than 15 years of ART had a progressive increase in the CD4:CD8 ratio without reaching a plateau. Factors associated with a persistently low CD4:CD8 ratio included older age, low nadir CD4 T cell count and detectable HIV plasma VL. No association was found between low CD4:CD8 ratio, HIV clade, co-receptor tropism, or co-infections with CMV, hepatitis B or hepatitis C viruses [30]. Interestingly, the metabolic status of treated patients having diabetes and/or hypertriglyceridemia was also linked with low CD4:CD8 ratio [30].

Cumulatively, these studies indicate that CD4 T cell count loses its predictive value, whereas the CD4:CD8 ratio remains a predictor of non-AIDS-associated morbidity and mortality even after long-term ART.

Recently, two groups reported results concerning the contribution of the CD4:CD8 ratio in prediction of cardiovascular events, the most frequent non-AIDS condition. Menozzi *et al*. performed a study on 914 patients receiving ART for over two years, and a threshold CD4:CD8 ratio value of 0.8 was chosen as a median value of the cohort [54]. In multivariable analyses, CD4:CD8 <0.8 was a predictor for risk of cardiovascular disease, and this effect was not evident for multimorbidity. Bernal *et al*. analyzed the associations between the CD4:CD8 ratio (≥1.0), cardiovascular risk factors and classes of ART drugs [55]. The inversion of the CD4:CD8 ratio in treated patients was independently associated with intimate media thickness, a marker of subclinical atherosclerosis. So far, there are no large-scale studies with sufficient statistical power to clearly assess the association between the CD4:CD8 ratio with other conditions, such as non-AIDS-associated malignancies, liver and kidney diseases.

### The CD4:CD8 ratio association with the size of the HIV reservoir

Viral rebound after cessation of long-term ART clearly indicates the existence of an HIV reservoir in long-lived infected cells [56,57]. Great efforts have been made to understand the mechanisms of viral latency, the size of the HIV reservoir and to design potential therapeutic approaches to target the latency [58,59].

Chun *et al*. explored the correlation between the CD4:CD8 ratio and HIV reservoir size [60]. They found an inverse correlation between the frequency of CD4 T cells carrying HIV-1 proviral DNA and the CD4:CD8 ratio in treated patients. Our group extended this study and confirmed that CD4 T cell nadir, the CD4:CD8 ratio and CD4 T cell counts were also inversely associated with HIV-1 proviral DNA levels. However, in multivariate analysis, only CD4 T cell nadir significantly predicted levels of HIV-1 proviral DNA independently of other factors [61].

The viral reservoir in peripheral blood exists predominantly in memory CD4 T cells endowed with reproductive potential, including central memory T cells (TCM) and transitional memory T cells (TTM). TCM are characterized by a high clonogenic potential and a relatively long half-life, whereas TTM represent an intermediate differentiation stage between TCM and effector memory T cells (TEM) [62,63]. Cockerham *et al*. found that the CD4 and CD8 T cell activation as measured by the expression of CD38, HLA-DR, CCR5 and PD-1, was associated with HIV DNA in resting CD4+ T cells in long-term ART-treated patients [64]. Based on these findings, CD4:CD8 ratio can be considered to reduce bias in patient allocation for clinical trials aiming at reducing the size of HIV reservoir [65].

### The impact of ART initiation on the normalization of CD4:CD8 ratio

The CD4:CD8 ratio normalization depends on several factors, such as level of immune activation, imbalance of the gut mucosa and the timing of ART initiation. From recent work, it appears that ART initiation in the early phases of infection allows for greater recovery of the CD4:CD8 ratio. Hocqueloux *et al*. reported on CD4:CD8 ratio recovery in a cohort where 35 patients began treatment during the first four months of infection, while 272 began later on in the chronic phase [66]. The early-treated group had a more rapid and sustained immune reconstitution and a CD4:CD8 ratio of 1.31 versus 0.77 in chronic patients (*p*<0.0001). A recent report by Thornhill *et al*. confirmed these findings in a prospective observational study on 353 patients, 253 of whom began early treatment while the remainder deferred ART initiation [67]. In early treated patients 45% normalized their CD4:CD8 ratio compared to only 11% in the deferred group. We further reported that ART initiated in the first year of infection normalized KT ratio and immune activation but failed to improve markers of gut mucosal dysfunction [42]. As of today only early ART initiation has been reported to be able to normalize CD4:CD8 ratio indicating the importance of further investigation.

### Future directions of research on the CD4:CD8 ratio

Many questions have yet to be answered. Relationship of the CD4:CD8 ratio and non-AIDS events may not be identified due to the lack of statistical power in some studies and deserves further evaluation. Therefore, virologically suppressed patients with low CD4:CD8 ratio should be monitored through long-term cohorts. Meanwhile, additional investigations on a large scale of patients with non-AIDS-associated cancer and neurocognitive impairment, as well as the impact of ART drug classes like integrase inhibitor are necessary. Association between the CD4:CD8 ratio and levels of microbial translocation, viral co-infections particularly CMV, needs to be further explored. Continued investigation on monocytes, innate immunity and immune-metabolism may also be helpful in elucidating the CD4:CD8 trajectory in treated HIV infection. Research should also focus on mechanisms associated with persistent CD8 T cell expansion and its impact on clinical outcome.

## Conclusions

Collectively, a persistently low CD4:CD8 ratio during long-term effective ART represents a marker of continuing immune dysfunction, “inflammaging” and high risk of non-AIDS morbidity and mortality. In long-term treated patients, the progressive correction of the CD4:CD8 ratio is solely a result of CD4 recovery, as CD8 T cell counts remains constant. Encouragingly, earlier ART initiation contributes to a more rapid CD4:CD8 ratio normalization when compared to late treatment initiation. However when ART is initiated in chronic phase, a moderate increase in the CD4:CD8 ratio is observed. Based on patient clinical outcome CD8 T cell count normalization should become a focus of research.

Overall, recent developments highlight the importance of CD4:CD8 ratio as a new tool for assessing patient clinical outcomes and response to immune-based therapies in the context of treated HIV infection.
